# The β-Grasp Domain of Proteasomal ATPase Mpa Makes Critical Contacts with the Mycobacterium tuberculosis 20S Core Particle to Facilitate Degradation

**DOI:** 10.1128/msphere.00274-22

**Published:** 2022-08-22

**Authors:** Xiansha Xiao, Xiang Feng, Jin Hee Yoo, Amanda Kovach, K. Heran Darwin, Huilin Li

**Affiliations:** a Department of Structural Biology, Van Andel Institute, Grand Rapids, Michigan, USA; b Department of Microbiology, New York University School of Medicine, New York, New York, USA; University of Maryland Medical Center

**Keywords:** ATPase, cryo-EM, Mycobacterium tuberculosis, proteasome

## Abstract

Mycobacterium tuberculosis possesses a Pup-proteasome system analogous to the eukaryotic ubiquitin-proteasome pathway. We have previously shown that the hexameric mycobacterial proteasome ATPase (Mpa) recruits pupylated protein substrates via interactions between amino-terminal coiled-coils in Mpa monomers and the degradation tag Pup. However, it is unclear how Mpa rings interact with a proteasome due to the presence of a carboxyl-terminal β-grasp domain unique to Mpa homologues that makes the interaction highly unstable. Here, we describe newly identified critical interactions between Mpa and 20S core proteasomes. Interestingly, the Mpa C-terminal GQYL motif binds the 20S core particle activation pocket differently than the same motif of the ATP-independent proteasome accessory factor PafE. We further found that the β-hairpin of the Mpa β-grasp domain interacts variably with the H0 helix on top of the 20S core particle via a series of ionic and hydrogen-bond interactions. Individually mutating several involved residues reduced Mpa-mediated protein degradation both *in vitro* and *in vivo*.

**IMPORTANCE** The Pup-proteasome system in Mycobacterium tuberculosis is critical for this species to cause lethal infections in mice. Investigating the molecular mechanism of how the Mpa ATPase recruits and unfolds pupylated substrates to the 20S proteasomal core particle for degradation will be essential to fully understand how degradation is regulated, and the structural information we report may be useful for the development of new tuberculosis chemotherapies.

## INTRODUCTION

Proteasomes are self-compartmentalizing proteases that play pivotal roles in protein homeostasis, stress response, protein quality control, and the regulation of various cellular processes (1–4). Proteasomes are widely found in eukaryotes and archaea, and occasionally in bacteria (5, 6). Proteasome structure is highly conserved: a 20S core particle (20S CP) is composed of four heptameric rings stacked axially to form a barrel (7). Eukaryotic proteasomes are activated at one or both ends by a 19S cap, which contains at least 19 different proteins, including an ATPase hexamer formed by Rpts 1 to 6 (2, 8). The eukaryotic 19S cap recognizes a covalently attached protein degradation signal called ubiquitin (Ub) on doomed proteins, which are unfolded and translocated in an ATP-dependent manner into 20S CPs, where polypeptides are hydrolyzed into oligopeptides (3, 9, 10). Model experimental substrates can also be unfolded by the archaeal proteasome-activating nucleotidase (PAN) (11, 12). Proteasomes can also be activated by the C termini of non-ATPase regulators such as the mammalian PA28 and the invertebrate PA26 complexes, although they share little sequence homology with each other (13, 14).

**FIG 1:**
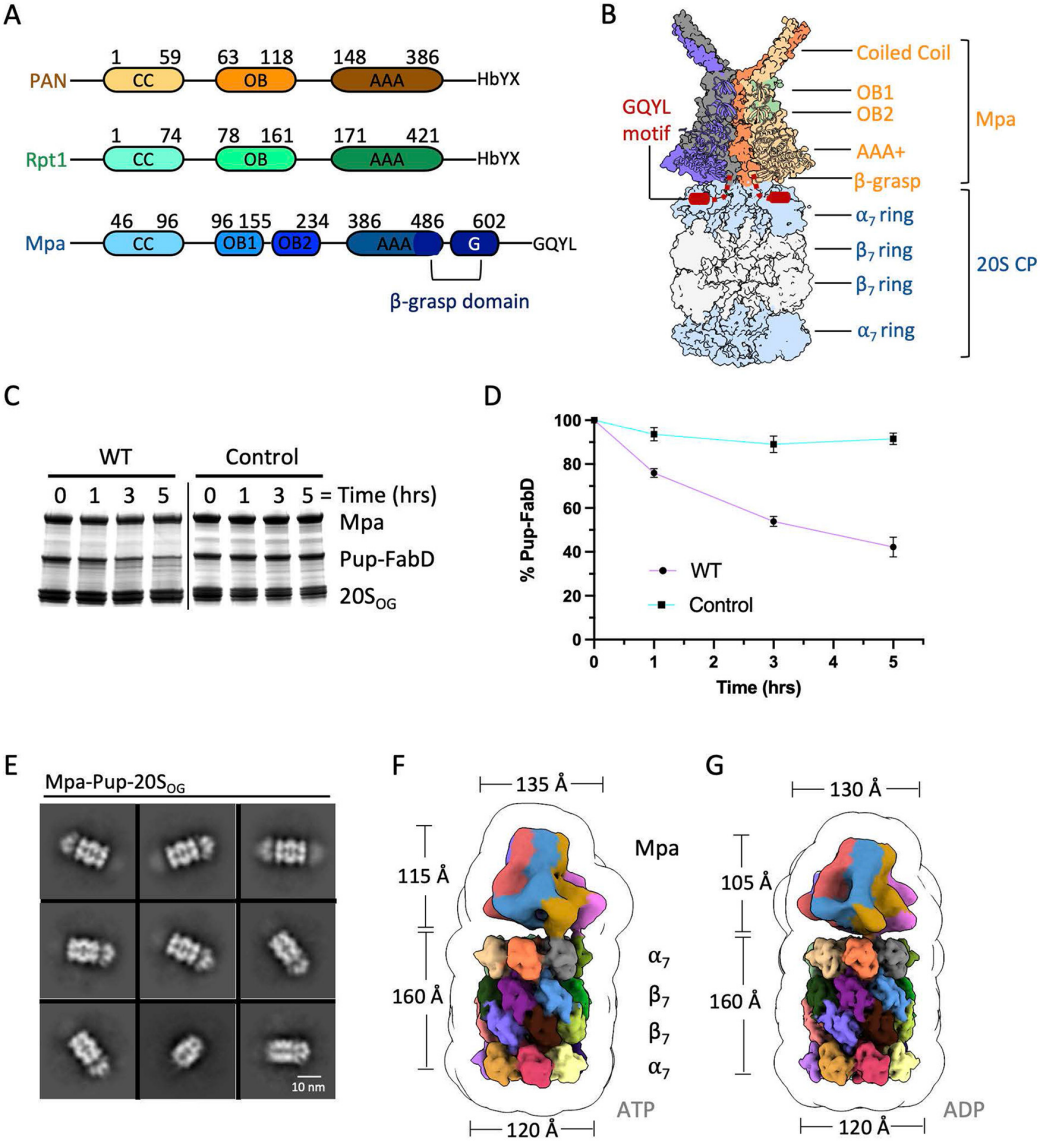
*In vitro* assembly and cryo-EM analysis of the M. tuberculosis Pup/Mpa/20S core particle system. (A) Domain architecture of Mpa, archaeal PAN, and human Rpt1. Mpa possesses the unique β-grasp domain. (B) A hypothetic computational model of the Mpa/20S CP complex. (C) Purified Pup-FabD was incubated with Mpa and 20S_OG_ CPs, or with denatured Mpa. Substrate degradation was monitored at indicated time points by SDS-PAGE. (D) Quantification of the remaining substrate by densitometry of the gel band using Image J. (E) Selected 2D class averages of Pup/Mpa/20S_OG_ CP in the presence of AMPPNP. (F to G) Surface rendering of the cryo-EM maps of the Mpa/20S_OG_ CP complex showing the full map embedded in a low-pass filtered 3D envelope in the presence of ATP (F) and ADP (G).

Mpa (known as ARC in nonmycobacterial species) recognizes prokaryotic ubiquitin-like protein (Pup) that is covalently attached to proteins destined for degradation by bacterial 20S CPs (15–18). The Pup-proteasome system in Mycobacterium tuberculosis is critical for bacteria to resist nitric oxide stress and is required to cause lethal infections in mice (17–19). Mpa structurally resembles the archaeal proteasome activator PAN and the eukaryotic Rpt1-6 ATPase ring; Mpa has a disordered amino (N)-terminal tail, followed by a coiled-coil domain, an intermediate oligonucleotide/oligosaccharide-binding (OB) domain, and a canonical AAA (ATPases associated with diverse cellular activities) domain (Fig. 1A and B) (20–22). Similar to the carboxyl (C)-terminal “hydrophobic-Tyr-any amino acid” (HbYX) motif of PAN and the Rpts needed for the activation of 20S CPs (23–25), Mpa bears a C-terminal “Gly-Gln-Tyr-Leu” (GQYL) motif for 20S CP gate opening (18). Interestingly, M. tuberculosis also encodes a non-ATPase proteasomal activator PafE (also known as Bpa) with the same C-terminal GQYL motif (26, 27). Unlike Mpa, PafE assembles into a dodecameric ring structure (28) that inserts its C termini into the activation pockets of M. tuberculosis proteasomes (29, 30). Importantly, PafE can robustly stimulate degradation of native substrates *in vitro* in the absence of ATP or other cofactors (27, 30).

Despite the resemblance to PAN and eukaryotic Rpts 1 to 6, Mpa contains several unique features: (i) an N-terminal coiled-coil that directly binds pupylated substrates (31); (ii) two rather than a single OB domain as found in PAN and Rpts 1 to 6 (22, 31); and (iii) a β-grasp domain near the C-terminus (32). Unlike Rpts 1 to 6 that bind robustly with 20S CPs, Mpa binds very weakly to its cognate 20S CPs *in vitro*, making it difficult to study their interaction (22, 33). Therefore, it is unknown how Mpa interacts and functions with 20S CPs for protein degradation in M. tuberculosis. In the ADP-bound crystal structure, Mpa assembles into symmetrical hexamers, and the β-grasp domain forms a constriction around a central channel that appears to inhibit rather than promote interactions with 20S CPs (32). However, Mpa in solution in the presence of ATPγS forms a flexible open spiral structure, with two of the six AAA domains unresolved by cryo-electron microscopy (EM) three-dimensional (3D) reconstruction (33). The open structure likely facilitates the initial engagement of Mpa with 20S CPs. Previous studies demonstrated that Mpa interacts poorly with the wild type 20S CP, but Mpa binds much better to an open gate 20S CP in which the N-terminal octapeptide of the α-subunit is removed (33, 34). Therefore, we used the open gate 20S CP (20S_OG_) in this study to obtain a stable complex of Mpa–proteasome. While this manuscript was under preparation, a high-resolution cryo-EM structural analysis of the M. tuberculosis Mpa-proteasome complex was reported, with the β-grasp domain making contacts with 20S CPs (35). In our work, we confirm the importance of the β-grasp domain in Mpa binding to 20S CPs. We further show using cryo-EM, *in vitro* degradation assays, and *in vivo* genetic mutants that the Mpa β-grasp domain interacts directly with the H0 helix of the 20S CP α ring, and that this interaction is essential for Mpa-mediated proteolysis.

## RESULTS

### Cryo-EM analysis of Pup or Pup-FabD in complex with Mpa and 20S_OG_ core particles.

We first confirmed the *in vitro* Pup-FabD degradation by purified Mpa and 20_OG_ (Fig. 1C and D). We then performed cryo-EM on *in vitro* assembled and gel-filtration purified Pup/Mpa/20S_OG_ or Pup-Fab/Mpa/20S_OG_ CP complexes in the presence of either ATP or ADP. In the two-dimensional (2D) class averages of the quaternary complex of Pup-Fab/Mpa/20S_OG_ CP purified by gel filtration, there were few structural features beyond the 20S CP (Fig. S1A to D). While this result was unexpected, we suggest that the tandem association between Pup-FabD and Mpa and between Mpa and 20S CP may have rendered these components too flexible to be detected by 2D classification and averaging. However, in the 2D averages of the Pup/Mpa/20S_OG_ CP complexes in the presence of ATP or ADP, structural features beyond the 20S_OG_ CPs were visible (Fig. 1E; Fig. S2A and B). We found that about 50% of the 20S_OG_ CPs were complexed with Pup/Mpa in the presence of ATP, compared to about 40% in the presence of ADP (Fig. S3 and 4).

10.1128/msphere.00274-22.3 FIG S1 Purification and cryo-EM of the Mpa/Pup-FabD/20S_OG_ CP complex. (A) Gel filtration profile of Mpa/Pup-FabD/20S_OG_ CP. (B) SDS-PAGE shows the presence of Mpa, Pup-FabD, and 20S CP in the gel-filtration peak. (C) A representative cryo-EM micrograph of gel-filtration peak of Mpa/Pup-FabD/20S_OG_ CP complex. (D) Selected 2D class averages showing the absence of Mpa and Pup-FabD densities on top of the 20S CP particles. Download FIG S1, TIF file, 0.6 MB. Copyright © 2022 Xiao et al. 2022 Xiao et al. https://creativecommons.org/licenses/by/4.0/ This content is distributed under the terms of the Creative Commons Attribution 4.0 International license.

10.1128/msphere.00274-22.4 FIG S2 Cryo-EM of the Mpa-Pup-20S_OG_ complex in the presence of ADP. (A) A representative cryo-EM micrograph of Mpa/Pup/20S_OG_ CP complex. Selected complex particles are highlighted in the red rectangular box. (B) 2D class averages of Mpa/Pup/20S_OG_ CP. Download FIG S2, TIF file, 0.4 MB. Copyright © 2022 Xiao et al. 2022 Xiao et al. https://creativecommons.org/licenses/by/4.0/ This content is distributed under the terms of the Creative Commons Attribution 4.0 International license.

10.1128/msphere.00274-22.5 FIG S3 Workflow for data processing of the cryo-EM images of the Mpa/Pup/20S_OG_ CP (ATP) complex. The first focused refinement step removed the unbounded or poorly bounded Mpa and the 20S_OG_ CP alone particles. In the final 3.2-Å resolution 3D map, the 20S CP region was well resolved, but the flexibly bound Mpa was not resolved. The Mpa density was resolved at 13-Å resolution through subsequent particle image down-sampling and focused refinement. Download FIG S3, TIF file, 0.7 MB. Copyright © 2022 Xiao et al. 2022 Xiao et al. https://creativecommons.org/licenses/by/4.0/ This content is distributed under the terms of the Creative Commons Attribution 4.0 International license.

We determined two composite 3D maps of the ternary complexes, one in the presence of ATP and the other in the presence of ADP (Fig. 1F to G; Table S2; Fig. S3 and 4). The flexible Mpa region was derived by masking and signal subtraction. The composite maps had an overall resolution of 3.2 Å and 3.0 Å, respectively, in the 20S_OG_ CP region, but were at a much lower resolution of 12 to 13 Å in the Mpa region. To explore the potential Mpa movement, we down-sampled the particle images and performed 3D variability analysis (36). In the resulting ensembles of 20 reconstructions numbered 0 to 19, we can see that while the position and conformation of 20S_OG_ CPs remained the same, those of Mpa varied (Fig. S5A and B). In the presence of ATP, all six AAA domains of the Mpa hexamer were flexible with weak densities. These domains were more stable in the presence of ADP with three or more AAA domains stabilized (Fig. S5B and C).

10.1128/msphere.00274-22.1 TABLE S1 Bacterial strains, plasmids, and primers used in this work. Download Table S1, DOCX file, 0.02 MB. Copyright © 2022 Xiao et al. 2022 Xiao et al. https://creativecommons.org/licenses/by/4.0/ This content is distributed under the terms of the Creative Commons Attribution 4.0 International license.

10.1128/msphere.00274-22.7 FIG S5 Mpa binds highly flexibly to the M. tuberculosis 20S_OG_ CP. Twenty conformations of Mpa/Pup/20S_OG_ CP complex in the presence of ATP (A) or ADP (B) in their side and top views derived by 3D variability analysis. The red asterisks in (A) and the black rectangles in (B) label 3D maps with relatively well-resolved Mpa AAA domains. In panel B, the ADP-bound Mpa/Pup/20S_OG_ CP 3D maps are divided into five groups, where individual AAA domains are resolved in group 1, not resolved at all group 2, and partially resolved in groups 3 to 5. (C) Superimposition of 3D maps of classes 8, 14, and 11 highlighted in rectangles in B, showing the different positioning of the Mpa above the 20S CP. Download FIG S5, TIF file, 1.0 MB. Copyright © 2022 Xiao et al. 2022 Xiao et al. https://creativecommons.org/licenses/by/4.0/ This content is distributed under the terms of the Creative Commons Attribution 4.0 International license.

### Interactions between the Mpa C-terminal GQYL motif and the 20S_OG_ CP activation pocket differ from that of the PafE/20S CP complex.

**FIG 2:**
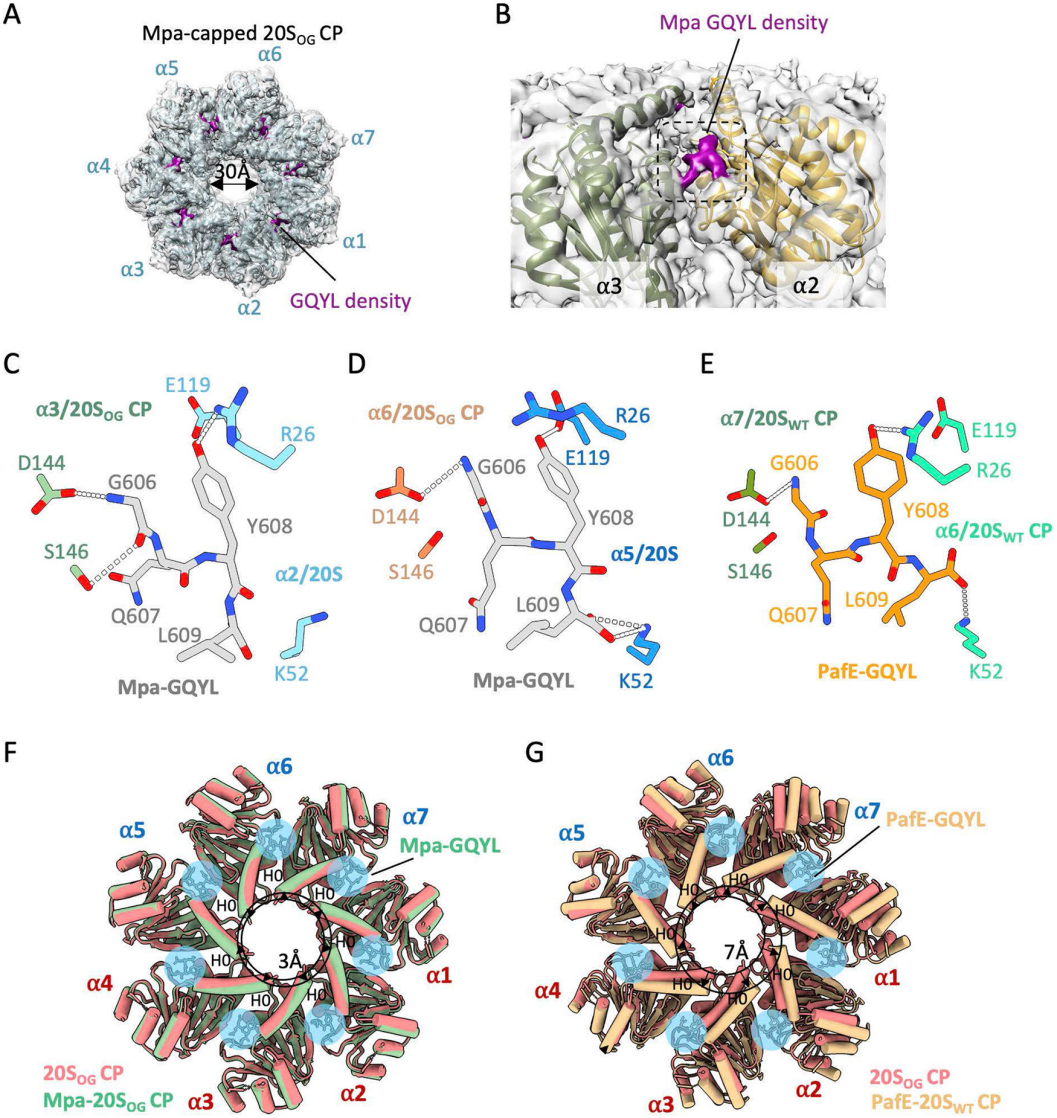
Two binding modes of the Mpa C-terminal GQYL in the M. tuberculosis 20S CP α-ring. (A) EM map of Mpa-bound 20S_OG_ CP α-ring at a high display threshold of 13σ. (B) EM density (magenta) of the GQYL motif at the interface of the α2 and α3 subunits. (C) The Mpa GQYL motif binding mode 1 observed in α1 to 4 and α7 and shown here with α2. (D) The Mpa GQYL motif binding mode 2 observed in α5 to 6 and shown here with α5. (E) The PafE GQYL motif in the previously reported structure of M. tuberculosis PafE/20S CP structure. H-bonds and salt bridges between Mpa GQYL and 20S CP α-rings are shown as dash lines. (F) Superimposition of the α-rings of 20S_OG_ CP alone (pink) and bound to Mpa (green) showing a 3 Å outward movement of the H0 helices. (G) Superimposition of the α-rings of 20S_OG_ CP alone (pink) and bound to PafE (yellow) showing an up to 7 Å outward movement of the H0 helices.

Mpa activates the M. tuberculosis 20S CP for protein degradation using a C-terminal GQYL motif (17), presumably by inserting the motif into the activation cavity in the proteasomal α-ring (37–39). But this assumption has not been directly examined for Mpa by a structural approach. In both high-resolution 3D maps at 3.1 to 3.3 Å, we observed a GQYL density inserted in each of the seven activation pockets in the α-rings of 20S_OG_ CPs (Fig. 2A and B). The presence of seven GQYL motifs of a hexameric Mpa complex was due to the averaging effect during 3D reconstruction of the 7-fold symmetric M. tuberculosis 20S CP. Unexpectedly, we observed two binding modes of the GQYL motif in the 20S_OG_ CPs. In the first binding mode observed in five α-subunits (α1 to α4, and α7), the penultimate Mpa Tyr608 hydrogen (H) bonds with Arg26 of α2, but the C-terminal Leu609 carboxylate does not form a salt bridge with α2 Lys52 (Fig. 2C). The GQYL motif was stabilized by two additional H-bonds with the adjacent α3 subunit: between Mpa Gly606 main chain nitrogen and carbonyl oxygen and α3 Asp144 and Ser146, respectively. In the second binding mode observed in the remaining two α-subunits (α5 to α6), the penultimate Mpa Tyr608 H-bonds with α5 Glu119, and the Leu609 carboxylate forms a lateral (horizontal) salt bridge with α5 Lys52, and the GQYL motif is further stabilized by an H-bond between the main chain nitrogen of Gly606 and the neighboring (α6) Asp144 (Fig. 2D).

PafE, the ATP-independent proteasome activator, shares no sequence homology with Mpa except for the C-terminal GQYL motif (residues 171 to 174 in M. tuberculosis PafE) (27). A previous cryo-EM analysis revealed the dodecameric ring structure and the detailed interaction between the PafE GQYL motif and the M. tuberculosis wild-type 20S CP (29, 30). Structural comparison shows that the GQYL motif of both activators uses the same pocket in the 20S CP α-ring. However, the specific interactions differ (Fig. 2C to E). In the PafE/20S CP structure with full length α-subunit, PafE Tyr173 forms a horizontal H-bond with α-subunit Arg26 (versus α5 Glu119 for Mpa Tyr608), and the Leu174 carboxylate forms a vertical salt bridge with α-subunit Lys52 (versus Mpa Leu609 carboxylate forming a horizontal salt bridge). The PafE GQYL is further stabilized by a H-bond between PafE Gly606 and Asp144 of the neighboring α-subunit.

The differential interactions of the GQYL motif of Mpa and PafE with the 20S CP are primarily caused by how deep the motif inserts into the α-ring activation pocket. The Mpa GQYL motif inserts 2 Å deeper in the first interaction mode and 4 Å deeper in the second interaction mode than the PafE GQYL motif. It is unclear if this difference is due to the intrinsic difference between PafE and Mpa, or due to the use of different CPs-open gate (with Mpa) versus wild type (with PafE) CPs in these studies.

In the M. tuberculosis 20S CP activation pocket, alanine substitution of Arg26 or Lys52 in the α-ring completely abolishes PafE-mediated degradation of the heat shock regulator HspR, indicating that both Lys52 and Arg26 are essential for M. tuberculosis proteasome function (28, 29). We next examined the importance of three remaining GQYL-interacting residues (Glu119, Asp144, and Ser146) in the M. tuberculosis 20S CP α-subunit for Mpa-mediated protein degradation. We individually substituted Glu119, Asp144, and Ser146 with alanine, purified the mutant 20S CP, and used the substrate Pup-FabD and wild type Mpa for *in vitro* protein degradation assays. Unexpectedly, the E119A mutant failed to degrade Pup-FabD. Because the 20S CP β-subunit band is shifted up to a position corresponding to the size of the uncleaved β-subunit in the SDS-PAGE (Fig. S6), we speculate that the mutation may have prevented the propeptide processing, leading to an inactive 20S CP such that no substrate degradation could occur. We found that both D144A and S146A substitutions slowed Pup-FabD degradation compared to the parental 20S_OG_ CP (Fig. S6). These results suggest that the interactions between the GQYL motif and the region of the activation pocket lined by residues of primary α-subunit are essential, but the region lined by residues of the neighboring α-ring seem to be less important.

10.1128/msphere.00274-22.8 FIG S6 Mpa promotes proteasomal degradation of pupylated substrate Pup-FabD by binding to the H0 helix on top of the 20S CP α-ring. Pup-FabD (10 μM) was incubated with Mpa (1 μM) and wild type 20S_OG_ CP (0.5 μM) or its α-subunit variants (E119A, D144A, S146A, 0.5 μM) in the presence of 5 mM ATP. The degradation progression is visualized at the indicated time points by Coomassie-staining SDS-PAGE. Note that the E119A mutation led to assembly of immature 20S CP in which the propeptide of the β-subunit remained uncleaved. Download FIG S6, TIF file, 0.2 MB. Copyright © 2022 Xiao et al. 2022 Xiao et al. https://creativecommons.org/licenses/by/4.0/ This content is distributed under the terms of the Creative Commons Attribution 4.0 International license.

We compared the dilation of the substrate entry pore by aligning the α-rings of the uncapped 20S_OG_ CP, the Mpa-capped 20S_OG_ CP, and the PafE-capped 20S full-length CP (Fig. 2F to G). These α-ring structures are highly similar, with an Cα RMSD of around 0.5 Å. We found that each of the seven α-subunits have rotated by 3° in the Mpa-bound form, leading to the H0 helix to move outward by approximately 3 Å. The H0 outward movement apparently account for the 30 Å open substrate entry pore of the Mpa-bound α-ring (Fig. 2A). A similar gate opening mechanism by GQYL-induced H0 dilation was observed by PafE (Fig. 2G). Thus, despite the differences in the residue-level interaction, the Mpa GQYL tail opens the 20S CP substrate entry with a similar mechanism as the PafE GQYL tail.

### Asymmetric binding between Mpa and 20S_OG_ CPs.

**FIG 3:**
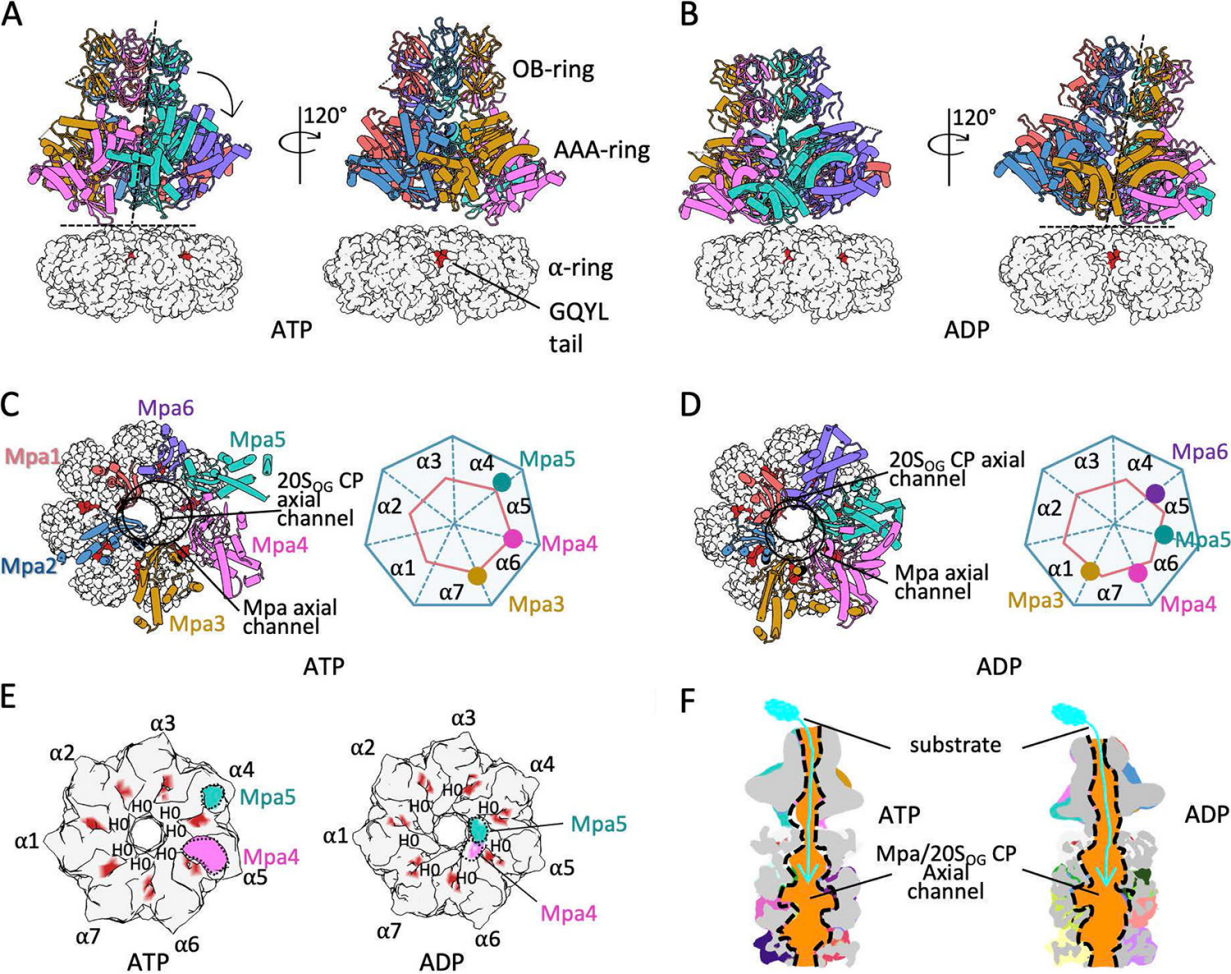
Representative structures of Pup/Mpa/20S_OG_ CP complexes. Overview of Pup/Mpa/20S_OG_ CP structure bound to ATP (A) or ADP (B). Only the top α-ring of 20S_OG_ CP (gray surface) is shown for simplicity. In both structures Mpa leans 5° to 10° toward 20S_OG_ CP as indicated by a dashed black line. (C and D). Top views of the Mpa/20S_OG_ CP showing the 20S CP binding Mpa AAA domains. The black circles mark the axial channels of the 20S CP and Mpa. The right panels illustrate the proximity of Mpa subunits to the 20S_OG_ CP α-ring. The pink hexagons and blue heptagons represent the six Mpa AAA domains and the seven 20S_OG_ CP α-subunits, respectively. The color dots represent Mpa AAA domains in close proximity to the 20S_OG_ CP α-ring. (E) Cut-open views of the interface between Mpa and the 20S_OG_ CP α–ring bound to ATP (left) and ADP (right). Red patches are the bound Mpa GQYL motif. (F) A vertically cut-open view showing the continuous substrate channel from Mpa axis to the 20S_OG_ CP proteolysis in the structure bound to ATP (left) or ADP (right).

We generated composite maps of the Mpa/20S_OG_ CP complex in the ADP-bound and the ATP-bound forms by combining the separately focus-refined Mpa and 20S maps. Mpa structure has been determined by X-ray crystallography in the ADP-bound form (32), by cryo-EM in the presence of ATP (33), and most recently by cryo-EM in complex with 20S_OG_ CPs in the presence of ATPγS (35). We found that the hexameric ADP-bound crystal structure best fit the ADP-bound composite map and the ATPγS-bound cryo-EM structure best fit the ATP-bound composite map (Fig. 3A and B). In both the ATP- and ADP-bound complexes, Mpa leans toward one side by 5° to 10°. The off-axis interaction was also observed between the archaeal PAN and its cognate proteasome (20). The interface between Mpa and a 20S_OG_ CP is mediated by the β-grasp domains at the bottom surface of Mpa. In the ATP-bound complex, three β-grasp domains (Mpa subunits 3, 4, and 5) contact the 20S CP α-ring, and in the ADP-bound complex (Fig. 3C), four β-grasp domains (subunits 3, 4, 5, and 6) are in proximity to the α-ring (Fig. 3D). However, two Mpa subunits (4 and 5) were primarily responsible for mediating the interaction in both structures (Fig. 3E). The substrate path from Mpa to the 20S CP was open and continuous in both structures (Fig. 3F).

### Contacts of the Mpa β-grasp domain with 20S CPs are required for protein degradation.

**FIG 4:**
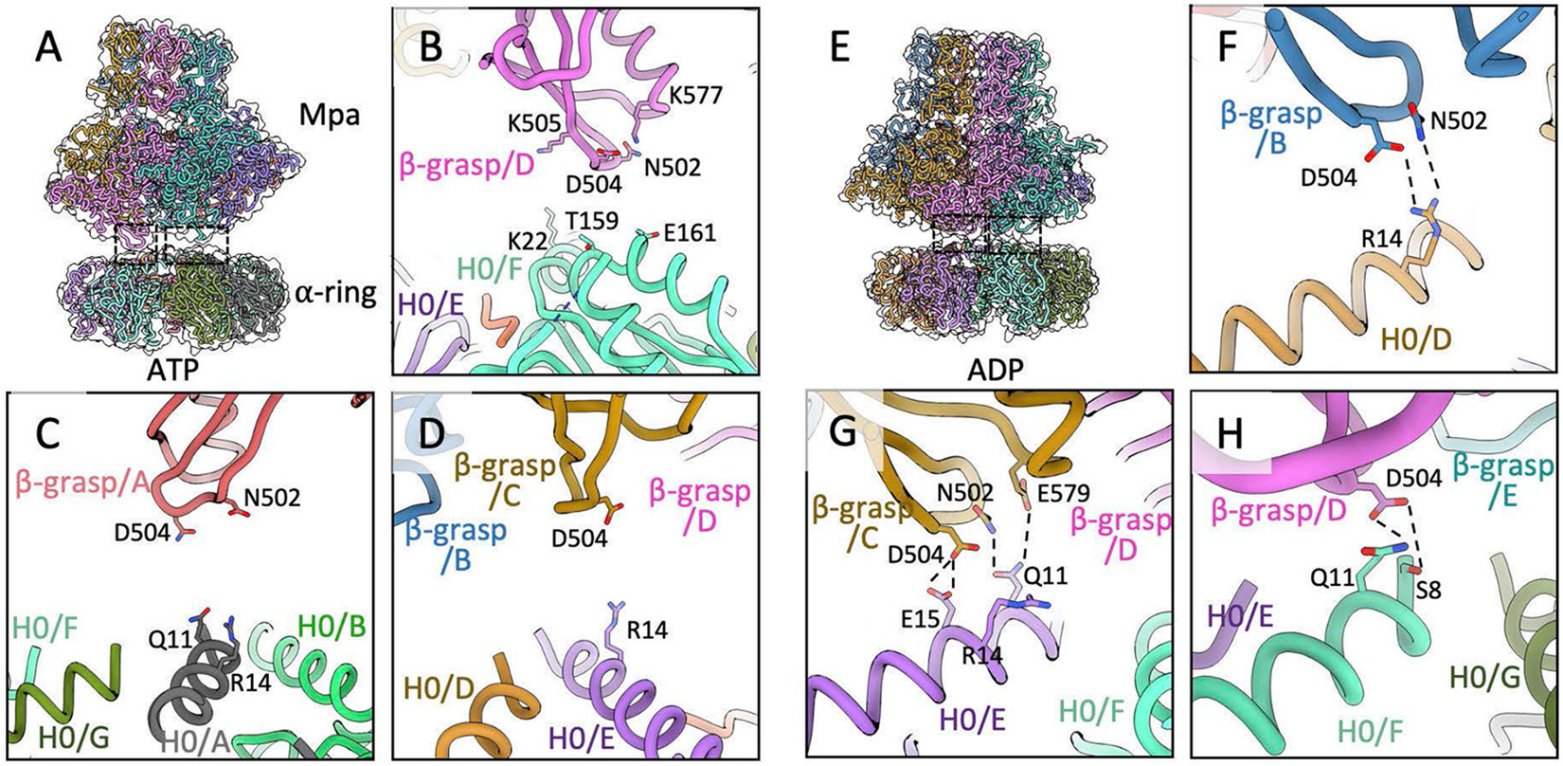
Detailed interactions between Mpa and 20S_OG_ CP. The interface of Mpa and the α-ring of 20S_OG_ CP in the presence of ATP (A to D) or ADP (E to H). (B to D) Close-up views showing the potential interacting residues between Mpa β-grasp domain and H0 helix of 20S_OG_ CP in the ATP bounded complex. (F to H) Close-up views showing the potential interacting residues between Mpa β-grasp domain and H0 helix of 20S_OG_ CP in the ADP bounded complex. Residues potentially involved in the interaction are shown in sticks. Dashed lines indicate likely hydrogen bonds.

The Mpa C-terminal β-grasp domain is composed of one helix (α13) and a three-stranded β-sheet (β16 to 18) (32). The structures of the Mpa/20S_OG_ CP complex show that a β-hairpin, composed of β-strands β16 to 17 of the β-grasp domain inserted in the helical subdomain of the AAA domain, primarily interacts with the top surface of the 20S CP α-ring. Specifically, the interaction involves residues Asn502, Asp504, and Glu506 in the Mpa β-hairpin and the H0-H3 helices of the 20S CP α-ring (Fig. 4A to H). The interactions differ in different nucleotide states and among different subunits. In the ADP-bound structure (Fig. 4F to H), Mpa Asp504 is within H-bond distance with Arg14, or with Ser8 and Gln11, or with Glu15 of the 20S CP H0 helix. In concert with Asp504, Asn502 may H-bond with Gln11 and Arg14, and Glu579 with Gln11 of the 20S CP H0 helix. Besides interacting with H0 helix, Asn502 may interact with the acidic Glu161 in the H3 helix in the ATP-bound structure (Fig. 4B and C). The multitude of interactions suggest that the Mpa β-grasp domain may rock alongside H0 during the binding-induced 20S CP gate opening and the subsequent ATP-hydrolysis-driven substrate translocation.

**FIG 5:**
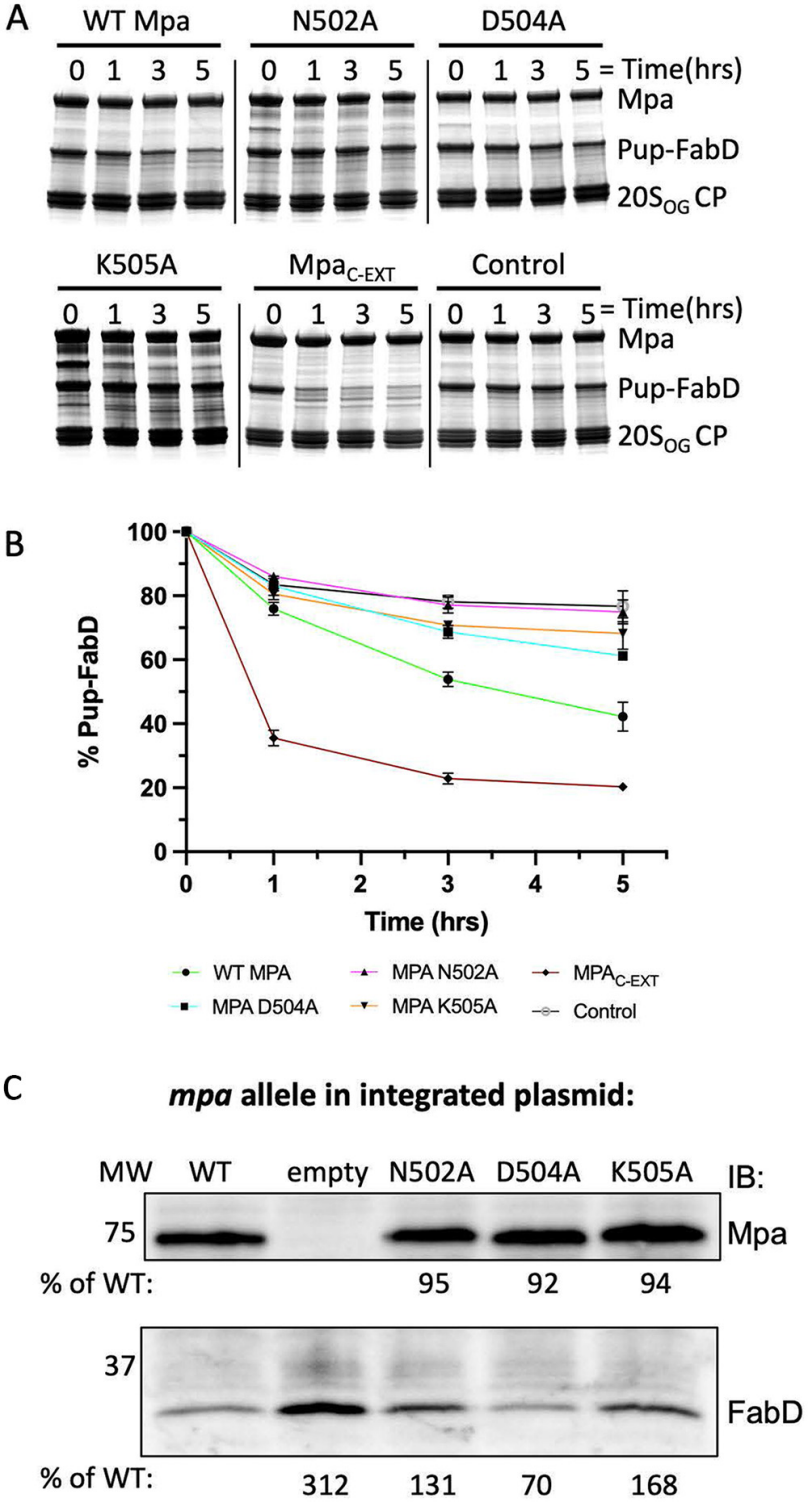
Mpa β-grasp domain promotes proteasomal degradation. (A) 10 μM Pup-FabD was incubated with purified wild type Mpa or its variants (N502A, D504A, K505A, Mpa_C-EXT_) and 20S_OG_ CPs in the presence of ATP. Control group was conducted the same way as in wild type Mpa except no nucleotide added. Degradation as monitored at the indicated time points by SDS-PAGE. (B) Quantification of the remaining substrate by densitometry of the gel bands using Image J. (C) Immunoblotting of M. tuberculosis lysates showed endogenous FabD accumulating in strains expressing Mpa_N502A_ and Mpa_K505A_. All strains are in the *mpa*::MycoMarT7 background and contained pMV306 with either wild type or mutant *mpa*. EV, empty vector. Antibodies to M. tuberculosis FabD were used. Blot is representative of two independent experiments. Quantification (% of WT) was performed using ImageJ.

To examine the importance of these residues in the Mpa β-grasp domain, we made three mutant Mpa proteins (N502A, D504A, and K505A) and assayed for their ability to mediate Pup-FabD degradation. As shown in Fig. 5A and B, all three mutant proteins exhibited a reduced ability to mediate substrate degradation compared to wild type Mpa or Mpa with a C-terminal six-residue extension (Mpa_C-EXT_) (32), both of which mediated Pup-FabD degradation by 20S_OG_ CPs. This result indicates that the interaction between the Mpa β-grasp domain and 20S CP is important for the *in vitro* function of the Pup-Mpa-proteasome system. We further examined if these Mpa residues were important for *in vivo* activity. We introduced the same three individual Mpa mutations N502A, D504A, and K505A in M. tuberculosis, and found that endogenous FabD accumulated in two of the three mutants (N502A, K505A) compared with bacteria with wild type Mpa, while the mutant D504A remained the same as wild type Mpa (Fig. 5C). It is unclear why the D504A mutation had little effect *in vivo*; but it is possible that *in vivo* other interactions mitigate the effect of this mutation. These results demonstrate the physiological relevance of the β-hairpin in mediating substrate protein degradation by 20S CPs.

## DISCUSSION

In this study, we used cryo-EM to understand how Mpa interacts with the 20S CP to mediate substrate protein unfolding and degradation. Our study has revealed three important insights: (i) the Mpa GQYL motif binds in a well-established activation pocket at the interface between two adjacent α-rings, but the binding mode can vary among the subunits; (ii) Mpa binding to the 20S CP is highly flexible and adopts different conformations, with the C-terminal β-grasp domain making multiple contacts with the top surface of the 20S CP α-ring; and (iii) Mpa interactions with 20S CPs are mediated by both the GQYL motif and the β-grasp domain, and both interactions are important for protein degradation. Our work complements the recently published high-resolution work by the Weber-Ban lab, which revealed Pup threading inside the ATPase chamber and a staircase-like, sequential peptide translocation mechanism (35).

The M. tuberculosis 20S CP α-subunit lacks the N-terminal reverse turn that is universally present in the archaeal and eukaryotic counterparts (14, 31, 40). Nevertheless, substrate entry is fully blocked by the seven chemically identical but conformationally distinct N-terminal peptides in the M. tuberculosis 20S CP (31). Gate opening of the M. tuberculosis 20S CP requires insertion of the C-terminal motifs of proteasomal activators into the CP activation pockets between two α-rings (41); because the C-terminal activation motif GQYL is identical in Mpa and PafE, it was unexpected that Mpa GQYL motif binds the 20S CP activation cavity differently from that of PafE, and there were two binding modes for the Mpa GQYL motif. We suggest that the different binding modes may reflect the different action mechanisms of the two activators, although we do not rule out the possibility that the use of the full-length versus the open gate CP may have caused the different binding. It is plausible that the GQYL motif of PafE stably binds in 20S CP activation pocket throughout the degradation of a protein substrate, whereas the GQYL motif may switch between the two observed binding modes or may dislodge from the activation pocket during Mpa-mediated substrate degradation, in response to the sequential ATP hydrolysis within the hexamer. This possibility is supported by our observation that the Mpa GQYL motif binds variably to the 20S CP activation pocket. Therefore, the secondary binding site in the β-hairpin of the Mpa β-grasp domain may have evolved to compensate for the transient and weaker binding of the Mpa GQYL motif in order to sustain the processivity of substrate threading by Mpa into the 20S CP.

The observed different binding of the β-hairpin to the 20S CP α-ring is likely an indication that a particular Mpa subunit undergoes a complex motion during the ATPase cycle of the hexamer. An Mpa subunit might slide on top of the 20S CP α-ring, interacting with the H0 helix at different radial locations, even interacting with the more peripherally located H3 helix. However, all these interactions are mediated by the β-hairpin of the β-grasp domain, in particular the five-residue segment (501-ANGDK-505) at the loop region of the β-hairpin. We individually mutated three of the five residues (N502A, D504A, and K505A) and found that two of these mutations noticeably reduced degradation of an endogenous substrate *in vivo*. Our result is consistent with the recent work demonstrating a significant decrease in protein degradation by Mpa with an altered five-residue segment (35). We suggest that these five residues of the β-hairpin loop collectively contribute to the interaction with the 20S CP.

In summary, our cryo-EM based analysis, together with the recently published work (35), has advanced our understanding of the Mpa ATPase-mediated protein degradation in M. tuberculosis. However, it is important to note that the biochemical pathway of the M. tuberculosis Pup-proteasome system is likely incomplete given that all studies to date have resorted to using open-gate 20S proteasomes to see robust degradation *in vivo*. Collectively these studies indicate that other factors are required for Mpa to interact with proteasomes, a hypothesis we are currently testing.

## MATERIALS AND METHODS

### Strains, plasmids, primers, and culture conditions.

Bacterial strains, plasmids, and primers used in this study are listed in Table S1. E. coli strains were grown in LB-Miller broth (Difco) with aeration on a roller drum or on LB agar at 37°C. M. tuberculosis strains were grown in Middlebrook 7H9 broth (Difco) supplemented with 0.2% glycerol, 0.05% Tween 80, 0.5% bovine serum albumin, 0.2% dextrose, and 0.085% sodium chloride. M. tuberculosis cultures were grown without shaking in 25 or 75 cm^2^ vented flasks (Corning) at 37°C. The antibiotics concentration used for M. tuberculosis growth were hygromycin at 50 μg/mL and kanamycin at 50 μg/mL. For E. coli hygromycin at 150 μg/mL was used.

To make the alanine mutations in Mpa for M. tuberculosis experiments, the residues of interest were mutated by splicing overlap extension (SOE) PCR (42) with Phusion polymerase, using pHD300 as the template. DNA oligonucleotides were purchased from either Invitrogen or Integrated DNA Technologies. SOE PCR products were clone into pHD300 using XhoI and ClaI sites. All enzymes were purchased from New England Biolabs. Calcium competent E. coli DH5α was transformed with ligations (43). The plasmids were sequenced by GENEWIZ, Inc. to confirm the veracity of their cloned sequences. Once the plasmids were confirmed, MHD5 was transformed with the plasmids by electroporation (44).

Mpa and Mpa_C-EXT_ were previously reported (32). For Mpa_C-EXT_, a five amino acid linker (GGGGS) was inserted between Thr601 and Glu602 of wild type Mpa. To produce a C-terminal intact protein for complex assembly with 20S_OG_ CPs, Mpa_C-EXT_ was produced with an internal 6 × His-tag inserted at Asp199. 20S_OG_ CPs were made with the N-terminal eight residues of α-subunits being deleted (31). Pup-FabD was made by fusing Pup to the N-terminus of FabD.

For purification of wild type Mpa and Mpa_C-EXT_, E. coli BL21(DE3) strains transformed with the respective plasmids were grown in LB medium supplemented with 50 μg/mL kanamycin at 24°C at an OD_600_ of 0.8. Temperature was lowered to 16°C and protein production was induced by isopropyl β-D-1-thiogalactopyranoside (IPTG) at a final concentration of 0.2 mM. After incubating overnight, cells were collected by centrifugation and resuspended in buffer A (20 mM Tris, pH 8.0, 200 mM NaCl, 2 mM MgCl_2_, 10% glycerol, 5 mM 2-mercaptoethanol). One EDTA-free protease inhibitor tablet (Roche) and 400 μL saturated phenylmethanesulfonyl fluoride (PMSF) (PMSF, Thermo Fisher) were added to the suspension before it was lysed by French press at 1,000 bars. After centrifugation at 36,600 × *g*, supernatant was loaded onto a 5 mL Ni^2+^-nitrilotriacetate acid (Ni-NTA) agarose column equilibrated with buffer A. Elution was conducted using buffer B (20 mM Tris, pH 8.0, 200 mM NaCl, 2 mM MgCl_2_, 10% glycerol, 5 mM 2-mercaptoethanol, 250 mM imidazole) after washing with 35 mM imidazole. Peak fractions containing Mpa_C-EXT_ was applied to a HiLoad 16/60 Superdex 200 pg gel filtration column in buffer C (20 mM Tris, pH 8.0, 200 mM NaCl, 2 mM MgCl_2_). Proteins were concentrated to 13.3 mg/mL and the purity was examined by SDS-PAGE. Pup-FabD and Mpa mutants (N502A, D504A, and K505A) were similarly purified.

M. tuberculosis 20S_OG_ CPs were purified as previously described (31). Briefly, 6 L of the appropriate E. coli strains were incubated at 37°C with shaking and induced with 0.3 mM IPTG when an OD_600_ reaches 0.7. For production of mature M. tuberculosis 20S_OG_ CPs, bacteria were induced at 37°C and grown overnight. The supernatant of lysed bacteria was purified by a 5-mL Ni-NTA column. Peak fractions of M. tuberculosis 20S_OG_ CPs were pooled, concentrated, and further purified by a HiLoad 16/60 Superdex 200 pg gel filtration column in buffer (10 mM HEPES, pH 7.5, 150 mM NaCl). 20S_OG_ CP mutants E119A, D144A, and S146A were purified similarly.

### *In vitro* degradation assay.

For wild type Mpa and Mpa_C-EXT_, degradation reactions were performed by incubating Pup-FabD (10 μM) with Mpa (1 μM) or Mpa_C-EXT_ (1 μM), and open-gate 20S CP (0.5 μM) at 30°C in buffer (25 mM Tris, 40 mM NaCl, 10 mM MgCl_2_, pH 8.0) supplemented with 10 mM MgCl_2_ and 1 mM DTT. Then, 5 mM ATP was added in the reaction mixture to initiate the reaction. At the indicated time points, aliquots were withdrawn from the reaction mixture and transferred into 2× concentrated SDS-PAGE sample buffer to terminate the reaction. All samples were analyzed by Coomassie brilliant blue-stained SDS-PAGE. The degradation assays for all mutant proteins (Mpa N502A, D504A, K505A, and 20S_OG_ CP E119A, D144A, S146A) were conducted similarly.

### Cryo-EM.

Purified Pup and Mpa were mixed at a final concentration of 1 μM and 4 μM, respectively, in buffer 10 mM HEPES, pH 7.5, 150 mM NaCl, 5 mM MgCl_2_, 1 mM DTT, 3 mM ATP or 10 mM HEPES, pH 7.5, 150 mM NaCl, 5 mM MgCl_2_, 1 mM DTT, 3 mM ADP. The mixture was incubated on ice for 1 h. Afterwards, 2 μM 20S_OG_ CP was added to the mixture and incubated on ice for another 1 h. Then, a 3-μL sample was applied to a holey carbon grid (Quantifoil Cu R1.2/1.3, 300mesh) that were glow-discharged for 30 s. The grids were blotted with a piece of Whatman 595 filter paper with the blot force set to 3 s and blot time set to 3 s. The blotted grids were flash-frozen in liquid ethane using FEI Vitrobot Mark IV with the chamber temperature set to 6°C and the humidity set to 95%. For ATP state, cryo-EM images were recorded with a Gatan K2 camera at a magnification corresponding to a pixel size of 0.603 Å in a Talos Arctica microscope (Thermo Fisher Scientific) operated at 200 kV. Thirty-frame movies were recorded with a dose rate of 0.2 electrons per Å^2^ per second and an exposure time of 6 s. For ADP state, the data set was collected in a FEI Titan Krios electron microscope operated at 300 kV high tension. A Gatan K3 summit direct detector positioned a GIF quantum energy filter was used. Data acquisition was performed with SerialEM under superresolution mode at a magnification of 130,000 and a pixel size of 0.431 Å per pixel. The defocus value ranges from −1.5 to −2.5 μm. The dose rate was 0.88 electrons per Å^2^ per second and the total exposure time was 1.5 s. The total dose was divided into a 60-frame movie, thus each frame was exposed for 0.03 s.

### Image processing and 3D construction.

We collected 1,840 raw movie micrographs for the Pup-Mpa/20S_OG_ CP-ATP complex and 11,189 raw movie micrographs for the Pup/Mpa/20S_OG_ CP-ADP complex. All the micrographs were drift corrected with electron-dose weighting and a binning factor of two by Motioncorr 2.1 (45). Contrast transfer function parameters of each aligned micrograph were calculated using CTFFIND 4.1 (46). For the data set of ATP state, after manual picking, some 600 particles from different views were obtained and further used for 2D class averages. Good 2D class averages were selected and used as templates for auto-picking, which generated 375,405 particles for 2D classification. 2D classes showing Mpa and 20S CP complex were selected and the particles from which were imported to cryoSPARC (version 3) for *ab initio* 3D model reconstruction (47). The model which contains all subunits was used as reference map for heterogeneous refinement. In the end, 213,000 particles containing all subunits were obtained, which were then converted to the RELION format by UCSF PyEM (https://github.com/asarnow/pyem). 3D refinement of these particles generated a 3D map of Mpa/20S CP complex. Mpa density was segmented in UCSF Chimera using Segger (48). Based on the Mpa density, a soft mask was created, which was then used for particle subtraction in RELION-3.0 (49). 3D classification was applied with the mentioned mask. Classes which show good Mpa features were selected for further focused 3D refinement. At the same time, particles corresponding to these classes were reverted to original particles and proceeded with 3D refinement. After postprocessing, CTF refinement, and Bayesian polishing, the final 3D density map reaches an average resolution of 3.2 Å as estimated by the gold-standard Fourier shell correlation at a correlation cutoff value of 0.143 (Fig. S3). The local resolution map was calculated using ResMap (50). To improve the Mpa density, the particles were down sampled by a factor of 4 (to 4.59 Å/pixel) and subjected to a 3D refinement. Particles from 3D refinement were then subjected to signal subtraction with a soft mask that include the Mpa or 20S CP region separately. A map was generated from PDB entry 7PXB and low pass filtered to 10 Å, which was used as reference for 3D refinement. The reference used for 3D refinement of 20S CP was generated similarly using PDB entry 6BGO. Refined Mpa and 20S CP regions were combined in ChimeraX (51).

For the data set of ADP state, template picking generated 2,102,567 particles for 2D classification. Good 2D classes were selected and a small part was used for Topaz training (52), which yielded 1,269,540 particles for 2D classification. Good 2D classes from template picking and Topaz training were combined, and duplicated particles were removed. An overall 1,285,028 particles were subjected for *ab initio* 3D model reconstruction in cryoSPARC (version 3), followed by heterogeneous refinement. A total of 512,694 particles were selected for the second round of *ab initio* 3D model reconstruction and heterogeneous refinement. In the end, 508,000 particles containing all subunits were obtained and refined to 3.0 Å as estimated by the gold-standard Fourier shell correlation (Fig. S4). Similarly, to improve the Mpa density, the particles were down sampled by a factor of 4 (to 3.31 Å/pixel) and subjected to a 3D refinement. Particles from 3D refinement were then subjected to signal subtraction with a soft mask that include Mpa or 20S CP region separately. A map was generated from PDB entry 5KZF and low pass filtered to 10 Å, which was used as reference for 3D refinement of Mpa density. The reference used for 3D refinement of 20S CP was generated similarly using PDB entry 6BGO. Refined Mpa and 20S CP regions were combined in ChimeraX.

10.1128/msphere.00274-22.6 FIG S4 Workflow for data processing of the cryo-EM images of the Mpa/Pup/20S_OG_ CP (ADP) complex. Topaz training was applied to increase the particle number. In the 3.0-Å resolution 3D map, 20S_OG_ CP was well resolved, but the Mpa region was poorly resolved. The EM density of the flexibly bound Mpa was captured at 12 Å resolution by image down sampling and focused 3D refinement. Download FIG S4, TIF file, 0.7 MB. Copyright © 2022 Xiao et al. 2022 Xiao et al. https://creativecommons.org/licenses/by/4.0/ This content is distributed under the terms of the Creative Commons Attribution 4.0 International license.

### Model building, refinement, and validation.

The modeling of 20S CP was based on the structure of PafE-20S (PDB 6BGO). Fourteen α-rings and 14 β-rings were directly docked as rigid bodies into the high-resolution EM map before down-sampling using Chimera. Mpa C-terminal loop was built in the density guided by the bulky residue Tyr. The model was subjected to manual adjustments in COOT (53), followed by real-space refinement in PHENIX (54). The final model was validated using MolProbity (55, 56). For modeling of Mpa density, the atomic model of Mpa was extracted from PDB entry 7PXB for ATP state and PDB entry 5KZF for ADP state, which were docked into the low-resolution Mpa and 20S CP EM map as rigid body, respectively. The refined 20S CP atomic model was also docked into the low-resolution Mpa and 20S CP EM map as rigid body, respectively. The combined model of Mpa and 20S CP was subjected to a real-space refinement in PHENIX. The final model was validated using MolProbity. Figures regarding to the 3D model were generated in either UCSF Chimera (48) and ChimeraX (57). Cryo-EM data collection, refinement, and validation statistics are listed in Table S2.

10.1128/msphere.00274-22.2 TABLE S2 Cryo-EM data collection, refinement, and validation statistics. Download Table S2, DOCX file, 0.02 MB. Copyright © 2022 Xiao et al. 2022 Xiao et al. https://creativecommons.org/licenses/by/4.0/ This content is distributed under the terms of the Creative Commons Attribution 4.0 International license.

### Immunoblotting for FabD in Mpa mutant M. tuberculosis strains.

M. tuberculosis cultures were grown to optical density at 580 nm (OD_580_) ~1.2 to 1.4. Bacterial densities were measured, and equivalent cell numbers were collected, based on the OD_580_ of the cultures. For example, an “OD_580_ equivalent of 1” indicates the OD of a 1 mL culture is 1.0. For most assays, 5 OD units were collected and washed once with phosphate-buffered saline with Tween 20 (PBS-T, recipe) to remove bovine serum albumin (BSA) in 7H9c media. Bacterial pellets were then resuspended in 300 μL TE buffer (100 mM Tris-Cl, 1 mM EDTA pH 8.0), and transferred to bead-beating tubes with 200 μL zirconia beads; tubes were beaten for 30 s three times, with icing for 30 s in between in a mini bead beater (all materials from BioSpec Products). Then, 150 μL of lysate was transferred into new tubes with 50 μL 4 × SDS sample buffer and boiled at 100°C for 10 min. Proteins were separated by 10% sodium dodecyl sulfate polyacrylamide gel electrophoresis (SDS-PAGE) and transferred onto nitrocellulose membranes. Membranes were blocked in 3% BSA prior to incubation with polyclonal rabbit antibodies. Mpa or FabD polyclonal antibodies were reported previously (17, 58). The immunoblot was developed with SuperSignal West Femto and imaged using a Bio-Rad ChemiDoc system. ImageJ was used for quantification of signal intensities (59).

### Data availability.

Cryo-EM 3D maps of the ATP-bound M. tuberculosis Mpa/20S_OG_ CP complex at 3.2 Å resolution and ADP-bound Mpa/20S_OG_ CP complex at 3.0 Å resolution have been deposited in the Electron Microscopy Data Bank under accession codes EMDB-27223 and EMDB-27224, respectively. The corresponding atomic models have been deposited in the Protein Data Bank under accession codes 8D6V and 8D6W, respectively. The two low-resolution composite structures in the ATP and ADP states also have been deposited under accession codes EMDB-27225 and PDB 8D6X and EMDB-27226 and PDB 8D6Y, respectively.
