# Supplementary material for: The β-Grasp Domain of Proteasomal ATPase Mpa Makes Critical Contacts with the Mycobacterium tuberculosis 20S Core Particle to Facilitate Degradation
Source: mSphere. 2022 Aug 22;7(5):e00274-22. doi: 10.1128/msphere.00274-22 (PMC9599533; doi:10.1128/msphere.00274-22)
Supplement: TABLE S1 [file msphere.00274-22-st001.docx]

|  | **Relevant genotype or description** | **Source or reference** |
| --- | --- | --- |
| **Plasmids:** | | |
| pET24b(+) | Kan^R^; for production of C-terminal His_6_ epitope-tagged protein | Novagen |
| pET24b(+)-His6-*pup* | Kan^R^; for overexpression of His_6_-*pup* | (1) |
| pET15b(+)-*mpa* | Amp^R^; for overexpression of mpa-His6 | (2) |
| pET24b(+)-*mpa* | Kan^R^; for overexpression of His6-*mpa_cext_* | (3) |
| pACYCDuet-*prcB*-his-*prcAΔN8* | Chl^R^; for overexpression of His6-*20s_og_* | (4) |
| pMV306 | Hyg^R^; plasmid that integrates to *M. tuberculosis* chromosome at *attB* site | (5) |
| pHD300 | Hyg^R^; plasmid that integrates to *M. tuberculosis* chromosome at *attB* site encoding WT *mpa* | (2) |
| pMV-*mpa*Asn502Ala | Same as pHD300 but with Asn502 mutated to Ala | This work. |
| pMV-*mpa*Asp504Ala | Same as pHD300 but with Asp504 mutated to Ala | This work. |
| pMV-*mpa*Lys505Ala | Same as pHD300 but with Lys505 mutated to Ala | This work. |
|  |  |  |
| **Strains** | | |
| OminiMax | F′[*proAB*^+^ *lacI*^q^ *lacZΔM15* Tn*10*(Tet^R^) Δ(*ccdAB*)] *mcrA* Δ*(mrr-hsdRMS-mcrBC)* φ80*(lacZ)ΔM15* Δ*(lacZYA-argF)U169* *endA1* *recA1* *glnV44* *thi-1* *gyrA96*(Nal^R^) *relA1* *tonA* *panD* | Novagen |
| BL21(DE3) | F^–^ *ompT* *gal* *dcm* *lon* *hsdS_B_*(*r_B_*^–^*m_B_*^–^) λ(DE3 [*lacI* *lacUV5*-*T7p07* *ind1* *sam7* *nin5*]) [*malB*^+^]_K-12_(λ^S^) | Novagen |
| DH5α | F^−^ ϕ80dlacZΔM15 Δ(lacZYA-argF)U169 deoR recA1 endA1 hsdR17(r_K_^−^ m_K_^+^) phoA supE44 λ thi-1 gyrA96 relA1 | Gibco, BRL |
| MHD5 | Kan^R^; *mpa*::MycoMarT7 | (6) |
| MHD22 | Kan^R^, Hyg^R^; same as MHD5 with pMV306 | (6) |
| MHD1689 | Kan^R^, Hyg^R^; same as MHD5 with pMV-*mpa*Asn502Ala | This work. |
| MHD1690 | Kan^R^, Hyg^R^; same as MHD5 with pMV-*mpa*Asp504Ala | This work. |
| MHD1691 | Kan^R^, Hyg^R^; same as MHD5 with pMV-*mpa*Lys505Ala | This work. |
|  |  |  |
| **Primers** | **Primer sequence (5’-3’)** | |
| MPA_N502A_fw | GACTTCCTTGTCGCCGGCGGCATAGGTGACCTCC | |
| MPA_N502A_rv | GGAGGTCACCTATGCCGCCGGCGACAAGGAAGTC | |
| MPA_D504A_fw | GTACATGACTTCCTTGGCGCCGTTGGCATAGGT | |
| MPA_D504A_rv | ACCTATGCCAACGGCGCCAAGGAAGTCATGTAC | |
| MPA_K505A_fw | CTTGAAGTACATGACTTCCGCGTCGCCGTTGGCATAGGTG | |
| MPA_K505A_rv | CACCTATGCCAACGGCGACGCGGAAGTCATGTACTTCAAG | |
| OG_K67A_fw | GTCGAACTCGTTGAACGCGCCCGCAGCCGCAAAA | |
| OG_K67A_rv | TTTTGCGGCTGCGGGCGCGTTCAACGAGTTCGAC | |
| OG_E119A_fw | CACACAACTCAACCGCGTAGGGCTTGGCC | |
| OG_E119A_rv | GGCCAAGCCCTACGCGGTTGAGTTGTGTG | |
| OG_D144A_fw | CGGCGATCGACCCGGCGTAGGTAATACGA | |
| OG_D144A_rv | TCGTATTACCTACGCCGGGTCGATCGCCG | |
| OG_S146A_fw | CGTCGGCGATCGCCCCGTCGTAGGT | |
| OG_S146A_rv | ACCTACGACGGGGCGATCGCCGACG | |
| OHD 72 pMV306seqR | CCTGTCGTTCACGGCTCTA | |
| OHD 98 mpa-int2-F | GTTCCTGCACAAGGAGTTGTACCG | |
| OHD 395 mpaAsn502F | GAGGTCACCTATGCCGCCGGCGACAAGGAAGTC | |
| OHD 396 mpaAsn502R | GACTTCCTTGTCGCCGGCGGCATAGGTGACCTC | |
| OHD 397 mpaAsp504F | CACCTATGCCAACGGCGCCAAGGAAGTCATGTAC | |
| OHD 398 mpaAsp504R | GTACATGACTTCCTTGGCGCCGTTGGCATAGGTG | |
| OHD 399 mpaLys505F | CCTATGCCAACGGCGACGCGGAAGTCATGTACTTC | |
| OHD 400 mpaLys505R | GAAGTACATGACTTCCGCGTCGCCGTTGGCATAGG | |

**References cited**

1. Pearce, M. J., Mintseris, J., Ferreyra, J., Gygi, S. P., and Darwin, K. H. (2008) Ubiquitin-Like Protein Involved in the Proteasome Pathway of <i>Mycobacterium tuberculosis</i>. *Science* **322**, 1104-1107

2. Yin, Y., Kovach, A., Hsu, H. C., Darwin, K. H., and Li, H. (2021) The mycobacterial proteasomal ATPase Mpa forms a gapped ring to engage the 20S proteasome. *J Biol Chem* **296**, 100713

3. Wu, Y., Hu, K., Li, D., Bai, L., Yang, S., Jastrab, J. B., Xiao, S., Hu, Y., Zhang, S., Darwin, K. H., Wang, T., and Li, H. (2017) Mycobacterium tuberculosis proteasomal ATPase Mpa has a β-grasp domain that hinders docking with the proteasome core protease. *Molecular Microbiology* **105**, 227-241

4. Wang, T., Darwin, K. H., and Li, H. (2010) Binding-induced folding of prokaryotic ubiquitin-like protein on the Mycobacterium proteasomal ATPase targets substrates for degradation. *Nature Structural & Molecular Biology* **17**, 1352-1357

5. Stover, C., De La Cruz, V., Fuerst, T., Burlein, J., Benson, L., Bennett, L., Bansal, G., Young, J., Lee, M., and Hatfull, G. (1991) New use of BCG for recombinant vaccines. *Nature* **351**, 456-460

6. Darwin, K. H., Ehrt, S., Gutierrez-Ramos, J. C., Weich, N., and Nathan, C. F. (2003) The proteasome of Mycobacterium tuberculosis is required for resistance to nitric oxide. *Science* **302**, 1963-1966
