# Supplementary material for: The β-Grasp Domain of Proteasomal ATPase Mpa Makes Critical Contacts with the Mycobacterium tuberculosis 20S Core Particle to Facilitate Degradation
Source: mSphere. 2022 Aug 22;7(5):e00274-22. doi: 10.1128/msphere.00274-22 (PMC9599533; doi:10.1128/msphere.00274-22)
Supplement: TABLE S2 [file msphere.00274-22-st002.docx]

|  | #1 **ATP state**  (EMDB-27223)  (PDB 8D6V) | #2 **ADP state**  (EMDB-27224)  (PDB 8D6W) | #1 **ATP state**  **composite**  (EMDB-27225)  (PDB 8D6X) | #2 **ADP state**  **composite**  (EMDB-27226)  (PDB 8D6Y) |
| --- | --- | --- | --- | --- |
| **Data collection and processing** |  |  |  |  |
| Magnification | 36,000 | 105,000 | 36,000 | 105,000 |
| Voltage (kV) | 200 | 300 | 200 | 300 |
| Electron exposure (e–/Å^2^) | 54 | 61 | 54 | 61 |
| Defocus range (μm) | 1.2-2.2 | 1.0-2.0 | 1.2-2.2 | 1.0-2.0 |
| Pixel size (Å) | 1.16 | 0.828 | 1.16 | 0.828 |
| Symmetry imposed | C1 | C1 | C1 | C1 |
| Initial particle images (no.) | 375,405 | 1,012,983 | 375,405 | 1,012,983 |
| Final particle images (no.) | 107,419 | 264,844 | 107,419 | 264,844 |
| Map resolution (Å)  FSC threshold | 3.2  0.143 | 2.9  0.143 | 13  0.143 | 12  0.143 |
| Map resolution range (Å) | 3.0-16.1 | 2.7-15.3 | 13-94 | 7.5-78 |
|  |  |  |  |  |
| **Refinement** |  |  |  |  |
| Initial model used (PDB code) | 6BGO | 6BGO | 6BGO  7PXB | 6BGO  5KZF |
| Model resolution (Å)  FSC threshold | 3.2  0.5 | 3.0  0.5 | -  - | -  - |
| Model resolution range (Å) | 3.0-16.1 | 2.7-15.3 | 13-94 | 7.5-78 |
| Map sharpening *B* factor (Å^2^) | -84 | -59 | - | - |
| Model composition  Non-hydrogen atoms  Protein residues  Ligands | 46380  6146  0 | 46380  6146  0 | 68362  8957  0 | 68262  8954  0 |
| *B* factors (Å^2^)  Protein  Ligand | 34.03  - | 40.11  - | 371.16  - | 296.98  - |
| R.m.s. deviations  Bond lengths (Å)  Bond angles (°) | 0.003  0.550 | 0.003  0.534 | 0.003  0.596 | 0.004  0.673 |
| Validation  MolProbity score  Clashscore  Poor rotamers (%) | 1.27  5.15  0 | 1.35  6.41  0.02 | 1.87  18.25  0.01 | 2.01  19.68  0 |
| Ramachandran plot  Favored (%)  Allowed (%)  Disallowed (%) | 98.16  1.84  0 | 98.36  1.64  0 | 97.47  2.53  0 | 96.58  3.35  0 |
